# ^99m^Tc-dextran lymphoscintigraphy can detect sentinel lymph node in breast cancer patients

**DOI:** 10.3892/etm.2014.2048

**Published:** 2014-11-04

**Authors:** LINLIN WEI, FANGNI CHEN, XUEHUI ZHANG, DANGSHENG LI, ZHONGQIANG YAO, LIYAN DENG, GUOYOU XIAO

**Affiliations:** 1 Department of Nuclear Medicine, Affiliated Cancer Hospital of Guangxi Medical University, Nanning, Guangxi 530021, P.R. China; 2 Graduate School of Guangxi Medical University, Nanning, Guangxi 530021, P.R. China; 3 Nuclear Medicine Department, Beihai People’s Hospital, Behai, Guangxi 536000, P.R. China

**Keywords:** lymphatic metastasis, sentinel lymph node biopsy, γ probe method

## Abstract

The aim of this study was to retrospectively determine the accuracy and feasibility of using ^99m^Tc-dextran (DX) lymphoscintigraphy for the localization of sentinel lymph nodes (SLNs) in breast cancer patients. The relevant factors affecting lymphoscintigraphy were also investigated. In this study, 235 breast cancer patients underwent ^99m^Tc-DX lymphoscintigraphic imaging and examination by a γ-probe method in combination with blue dye staining to detect SLNs. The detection results were considered in combination with rapid frozen pathology results to determine whether SLN metastasis was positive or negative. SLNs were identified in 191 patients by γ-probe detection among the 202 patients that tested positive by lymphoscintigraphic imaging, a coincidence rate of 94.6%. This suggested that lymph node metastasis had occurred and could be detected using lymphoscintigraphy. The axillary status of the breast cancer patients was also predicted using lymphoscintigraphy and the false-negative rate, sensitivity, specificity and positive predictive value were 13.3% (4/30), 90.7% (39/43), 23.4% (45/192) and 13.5% (21/155), respectively. The age of the patient, menstrual status, tumor location, tumor size, pathological type, preoperative biopsy and neoadjuvant chemotherapy were unrelated to the success of lymphoscintigraphy (P>0.05). ^99m^Tc-DX lymphoscintigraphy is able to exactly determine the location of SLN in breast cancer patients, and can be used for guiding γ-probe methods and sentinel lymph node biopsy.

## Introduction

Breast cancer is one of the most common cancers in females. The incidence of breast cancer ranks among the top two cancers in Chinese females, and is a serious threat to health (1). Lymphatic metastasis often occurs at the early stage of breast cancer, which is one of the prognosis factors and is key to determining the clinical stage and for guiding the selection of breast cancer treatment (2). Axillary lymph node dissection (ALND) in combination with pharmacology examination (99mTc-dextran lymphoscintigraphy) has been considered to be the most accurate method for evaluating lymphatic metastasis; however, ALND usually leads to a series of short- or long-term complications, such as wound infection, hematoma formation, pain and limitation of shoulder activity (3). Moreover, ALND is not significant in the diagnosis of early-stage breast cancer patients who are axillary lymph node-negative, but may seriously affect the patient’s quality of life (4). In the 1990s, the concept of sentinel lymph nodes (SLNs) was introduced into clinical practice. A number of studies have indicated that sentinel lymph node biopsy (SLNB) can predict axillary lymph node metastasis accurately (5,6).

In the treatment of breast cancer, SLNB is quite significant for the reduction of the upper extremity complications of patients and for the prediction of axillary lymph node state, and has gradually become an integral part of the comprehensive treatment of breast cancer. The identification and location of SLNs are the key findings of successful SLNB. Currently, the methods used for the identification and location of SLN include lymphoscintigraphy, blue dye methods, or a combination of the two methods.

This study retrospectively evaluated the accuracy of ^99m^Tc-dextran (DX) lymphoscintigraphy for the identification of SLN location in 235 consecutive cases of breast cancer in female patients, and analyzed relevant factors affecting the success of imaging.

## Materials and methods

### Patients

In this study, 235 consecutive cases of breast cancer in female patients diagnosed at the Affiliated Cancer Hospital of Guangxi Medical University (Nanning, China) from January 2009 to December 2012 were collected as the experimental subjects. All patients received lymphoscintigraphy prior to radical mastectomy at the Department of Nuclear Medicine of the Affiliated Cancer Hospital of Guangxi Medical University. The case inclusion criteria included the conditions as follows: i) female patients, ii) preoperative fine needle aspiration or biopsy and intraoperative frozen pathology verified breast cancer and iii) clinical stage T1–T2 phase patients. The case exclusion criteria were as follows (cases with any of the following were excluded from this study): i) patients receiving ipsilateral axillary trauma or previous surgery, ii) patients receiving ipsilateral breast cancer surgery, iii) pregnant or lactating patients and iv) patients with short-term relapse after radical mastectomy. Patients provided signed informed consent. Prior written and informed consent was obtained from every patient and the study was approved by the ethics review board of the Affiliated Tumor Hospital of Guangxi Medical University.

### Equipment and materials

^99m^Tc-DX and lyophilized dextran conjugate were provided by Beijing Senke Pharmaceutical Co., Ltd. (Beijing, China). The radiochemical purity by paper chromatography analysis was >90% and the marking rate was >95%, with a particle size of 50–200 nm. A molybdenum-technetium generator was provided by Beijing Atom Hi-Tech Co., Ltd. (Beijing, China). Single-photon emission computed tomography (SPECT) was achieved using a dual-head Discovery VH SPECT scanner purchased from (GE Healthcare, Pittsburgh, PA, USA), which was configured with a low-energy high-resolution collimator. The sensitive ray energy range of the Europrobe hand-held γ detector (Eurorad, Eckbolsheim, France) was 100–1,000 keV, and 1% methylene blue (MB) was provided by Beijing Yongkang Pharmaceutical Factory (Beijing, China).

### Preoperative lymphoscintigraphy for SLN location

**Figure 1:**
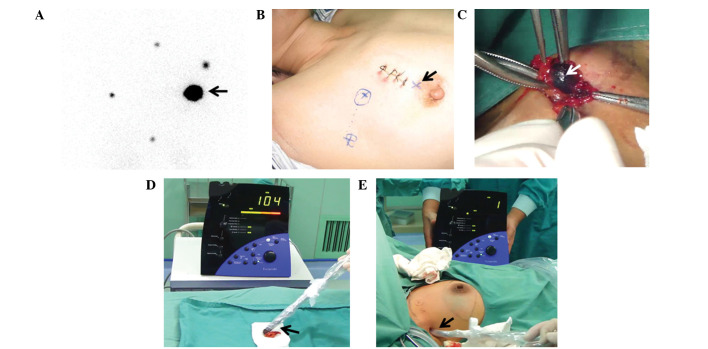
Examination of a breast cancer patient (A) The left axillary lymph node was shown to be positive by lymphoscintigraphy. Arrow indicates the subcutaneous tracer injection site outside the left areola (^99m^Tc-DX, 37–74MBq, (1–2mCi)/0.5–1.0 ml was injected). (B) The node location was marked on the body surface after imaging. Arrow indicates the subcutaneous tracer injection site on the edge of the right areola. (C) Blue stained lymph nodes were investigated by lymphoscintigraphy. Arrow indicates the detected blue-stained lymph nodes. (D) Whether the blue-stained lymph nodes were hot nodes was determined. Arrow indicates γ detector probe. (E) Probe following the removal of the lymph node. Arrow indicates the surgical site detected again by γ-detector probe.

Within 14–17 h prior to conducting the surgery, ^99m^Tc-DX 37–74 MBq (1–2 mCi)/0.5–1.0 ml, was injected at sites including the subcutaneous area the tumor site, the mammary areola and the area surrounding the biopsy residual cavity. Lymphoscintigraphy was performed following the injection at 15 min, 30 min, 1 h and 2 h, and at 1 h prior to surgery the next day. The SPECT acquisition conditions were set as matrix, 256×256; 1-fold magnification; peak energy, 140 keV; window width, 20%; and frame count, 5×10^5^. A freshly prepared ^99m^TcO_4_ point source was used as the location source, which included an active point source and four fixed-point sources located at the supraclavicular fossa, xiphoid, and bilateral areola. A collective focus of radioactive density, with the exception of that at the injection sites, was considered as positive lymph node imaging (Fig. 1A). Active point sources between the probe and imaging area helped to determine the surface location of the collective focus of density and were marked on the body surface (Fig. 1B).

### Intraoperative blue dye methods and ‘hot spot’ detection

As shown in Fig. 1C, during the surgery, the patient was maintained in a supine position and 1% MB was administered by subcutaneous injection. After 5 min, SLNB was performed along the blue-stained lymph vessels to detect the stained lymph nodes. At the same time, the handheld γ detector was used to detect hot nodules, which were those having a count 10-fold higher than the basal count. The counts of cold nodules were 10% of those of the hot nodules, and the counts of warm nodules were between those of the hot and cold nodules (Fig. 1D and E).

### SLN treatment and axillary treatment methods

All the blue-stained lymph nodes and warm nodules resected during the surgery were considered as SLNs and were sent for rapid frozen section pathological biopsy. If the results suggested that SLN metastasis had occurred, routine ALND was carried out. If no metastasis was identified, the surgeon decided whether the patient could be treated with ALND. However, the lymph node detection was considered to have failed if no blue-stained lymph nodes or hot nodules were detected, and if this occurred, the patients were treated with ALND.

### Evaluation criteria

The location accuracy of lymphoscintigraphy was evaluated on the basis of the criteria for evaluating SLNB (7). The following formulae were applied: i) γ-probe detection rate (%) = (SLN-positive cases/SLN cases detected) ×100; ii) sensitivity = (imaging-positive cases/SLN metastasis cases); iii) false negative rate (%) = (imaging-false negative cases/SLN metastasis cases) × 100; iv) specificity (%) = (imaging-true negative cases/SLN-negative cases + SLN-false positive cases) ×100; and v) positive predictive value = imaging-true positive cases/(SLN-true positive cases + SLN-false positive cases).

### Statistical analysis

SPSS statistical software, version 15.0 (SPSS, Inc., Chicago, IL, USA) was used to analyze the data, which were compared using the χ^2^ test. Differences were considered to be statistically significant when P<0.05.

## Results

### Lymphoscintigraphy

The clinical characteristics of the patients are shown in Table I. The ages of the enrolled patients ranged from 24 to 77 years old, with a median age of 45 years. There were 63 cases of patients who were menopausal, and 172 cases that were premenopausal. There were 140 patients that had tumors located in the upper outer quadrant, while the remaining 95 cases had tumors in other quadrants. A tumor size ≤2 cm was found in 123 cases, and there were 112 cases with tumors >2 cm but ≤5 cm. Moreover, there were 16 cases of ductal carcinomas *in situ*, 185 cases of invasive ductal carcinomas, 12 cases of invasive lobular carcinoma and 22 cases of other types, including squamous cell carcinoma and mucous adenocarcinoma.

In this study, 202 patients among the 235 patients showed positive results in lymphoscintigraphy imaging. The detection rate was 86.0% (202/235). Moreover, 191 cases of these 202 patients were detected to have ‘hot nodules’ or ‘warm nodules’. In addition, there were 11 cases of patients in which no SLNs were detected by lymphoscintigraphy prior to surgery or by γ-probe methods during the surgery, but in which SLNs were detected using blue dye methods. The detecting coincidence rate was 94.6% (191/202) for lymphoscintigraphy in combination with the γ-probe method. The successfulness of lymphoscintigraphy was identified to have no association with the age of the patient, menstrual status, tumor location, tumor size, pathological type, preoperative biopsy and neoadjuvant chemotherapy (P>0.05; Table I).

### Comparison of lymphoscintigraphy results with lymph node pathology results

A comparison of the results showed that there was one patient for which lymphoscintigraphic imaging gave a positive result prior to surgery, but in which blue stained lymph nodes or ‘hot spots’ were not observed during the surgery, while the pathology results following ALND found that lymph node metastasis had occurred (1/11). Moreover, there were 11 patients for whom negative results were obtained by lymphoscintigraphy prior to surgery, but blue-stained lymph nodes were detected during surgery. Intraoperative frozen section pathology and postoperative routine pathology confirmed that metastasis to the blue-stained lymph node had not occurred in these 11 patients, nor to the lymph nodes obtained from ALND. A further five patients (5/33) received negative lymphoscintigraphy imaging results and were not found to have SLNs during the surgery; however, following ALND, pathology results indicated that axillary lymph node metastasis had occurred.

### Lymph node pathology results and axillary treatment

Pathology results demonstrated that there were 43 cases of SLN-positive patients and 159 cases of SLN-negative patients, with 17 cases that were axillary node-positive and 215 cases that were axillary node-negative. A total of 55 patients received only SLN resection, and the intraoperative frozen pathology biopsy showed that all these 55 cases were SLN-negative. Thus, they were not treated with ALND. A total of 180 cases received ALND, and pathology results suggested that 23 of them were SLN-positive but axillary node-negative.

### Evaluation of the positioning accuracy of lymphoscintigraphy

Based on the evaluation criteria, the SLNB results obtained using ^99m^Tc-DX lymphoscintigraphy were analyzed. The detection rate of the γ probe in patients that were positive by lymphoscintigraphy was 94.6% (191/202), and the false negative rate for predicting axillary status was 13.3% (4/30). Moreover, the sensitivity was 90.7% (39/43), the specificity was 23.4% (45/192) and the positive predictive value was 13.5% (21/155). These results suggest that the use of the γ-probe method in lymphoscintigraphy can localize SLN accurately for SLNB, but is not suitable for use in the determination or prediction of axillary lymph node metastasis.

## Discussion

In the present study, lymphoscintigraphic imaging in combination with γ-probe analysis had an SLN detection rate of 94.6% (191/202), and the false negative rate for predicting axillary status was 13.3% (4/30). Moreover, the sensitivity was 90.7% (39/43), the specificity was 23.4% (45/192), and the positive predictive value was 13.5% (21/155). Due to the role of macrophage phagocytosis in lymph nodes, lymphoscintigraphic tracers were retained within the SLNs to enable the radiographic imaging. The lymph node contents as well as the distribution, shape, size and functional status of lymphatic vessels may be observed by lymphoscintigraphic imaging (8–11). Thus, this method can diagnose the lymph node metastasis of malignant tumors, and also determine pathological changes in the lymphatic system caused by a benign condition (12–14).

The detection rate of lymphoscintigraphy is ~90–97%, which is similar to that reported in the majority of literature (15,16), indicating that the use of a γ probe in lymphoscintigraphy may provide accurate SLN localization for SLNB. However, the SLN imaging of the patients conducted using lymphoscintigraphy is not fully developed. Statistical analysis demonstrated that the success of lymphoscintigraphy had no correlation with patient age, menstrual status, tumor location, tumor size, pathological type, preoperative biopsy and neoadjuvant chemotherapy (P>0.05). However, relevant factors include the injection site and the degree of lymph node invasion (17,18).

The enrolled patients were all treated with the same radioactive tracers with the same volume of injection dose and the same imaging conditions so that the success rate of lymphoscintigraphy was less affected by these factors (19–21). ^99m^Tc-DX lymphoscintigraphy is able to accurately locate breast SLNs to guide breast SLN location with the use of a γ probe and SLNB (22–24). However, individualized treatment for patients should be considered in order to improve the success rate of imaging and better guide SLNB.

## Figures and Tables

**Table I:** Clinical characteristics of patients and the associated lymphoscintigraphy results.

Clinical characteristics	Lymphoscintigraphy positive, n (%)	Lymphoscintigraphy negative, n (%)	χ^2^	P-value
Patient age (years)				
≤30	5 (83.3)	1 (16.7)	0.054	0.973
>30, ≤50	133 (85.8)	22 (14.2)		
>50	64 (86.5)	10 (13.5)		
Menstrual status				
Menopausal	52 (82.5)	11 (17.5)	0.491	0.483
Premenopausal	150 (87.2)	22 (12.8)		
Tumor size				
T1: ≤2 cm	108 (87.8)	15 (12.2)	0.730	0.393
T2: >2 cm, ≤5 cm	94 (83.9)	18 (16.1)		
Tumor location				
Upper outer quadrant	116 (82.6)	24 (17.4)	2.158	0.141
Other quadrants	86 (90.5)	9 (9.5)		
Preoperative biopsy				
Yes	136 (83.4)	27 (16.6)	2.162	0.141
No	66 (91.7)	6 (8.3)		
Neoadjuvant chemotherapy				
Yes	19 (95.0)	1 (5.0)	0.775	0.378
No	183 (85.1)	32 (14.9)		
Type of tumor				
Intraductal carcinoma	14 (87.5)	2 (12.5)	2.178	0.536
Invasive ductal carcinoma	157 (84.5)	28 (15.5)		
Invasive lobular carcinoma	12 (100)	0 (0)		
Others	19 (86.4)	3 (13.6)		
